# Effective recruitment strategies for home-living vulnerable older adults with depression into a psychotherapy RCT

**DOI:** 10.1093/geroni/igab046.3110

**Published:** 2021-12-17

**Authors:** Eva-Marie Kessler, Fee Hoppmann, Julie L O’Sullivan, Paul Gellert, Christina Tegeler

**Affiliations:** 1 MSB Medical School Berlin, Berlin, Berlin, Germany; 2 Charité – Universitätsmedizin Berlin, Charité – Universitätsmedizin Berlin, Berlin, Germany; 3 Charité – Universitätsmedizin Berlin, 10117, Berlin, Germany; 4 MSB Medical School Berlin, MSB Medical School Berlin, Berlin, Germany

## Abstract

**Objectives:**

Vulnerable older adults, such as physically impaired or care-dependent individuals, are vastly underrepresented in psychotherapy research. Improving their inclusion in randomized controlled trials is necessary to determine the effectiveness of psychotherapy in this population. This study is the first to systematically evaluate strategies to recruit home-living vulnerable older adults with clinically significant depression into a large randomized controlled psychotherapy trial. Potential participants were approached directly (self-referral) or via cooperation with gatekeepers (gatekeeper-referral).

**Methods:**

The initiator of the first contact with the study team and successful recruitment strategies were recorded. Referral strategies were compared with respect to number of inquiries and inclusion rates; study personnel’s time investment; and participant characteristics (sociodemographics, functional and cognitive status, depression and anxiety scores).

**Results:**

Most of the N=197 participants were included via gatekeeper-referral (80.5%, 95%CI=[74.9%,86.1%], but time investment for gatekeeper-referrals was five times higher than for self-referral by media reports. Clinical psychologists and medical practitioners referred the largest proportion of participants (32.3% each) and referral by medical practitioners led to highest inclusion rates (55.6%; χ²(3)=8.964, p<.05). Most participants were referred from a hospital setting (50.3%), whereas referral numbers by medical practices were low (15.9%). Participants who initiated the first contact themselves had higher inclusion rates and were less functionally and cognitively impaired.

**Conclusions:**

Including home-living vulnerable older adults into psychotherapy trials requires simultaneous implementation of diverse recruitment strategies. Medical practitioners and psychologists, especially in hospitals, are the most effective recruitment strategy, but self-referral via media is most cost-efficient in terms of time investment.

